# Duplication of the Gallbladder. A Case Report

**DOI:** 10.1155/2009/483473

**Published:** 2009-11-30

**Authors:** G. Desolneux, S. Mucci, J. Lebigot, J. P. Arnaud, A. Hamy

**Affiliations:** ^1^Service de Chirurgie Digestive et Endocrine, CHU Angers, 49933 Angers Cedex 9, France; ^2^Service de Radiologie, CHU Angers, 49933 Angers Cedex 9, France

## Abstract

Gallbladder duplication is a rare anatomic malformation, which can now be detected by preoperative imaging study. We report a case of a symptomatic duplicated gallbladder, successfully treated by laparoscopic cholecystectomy. This anomaly is important to know for surgeons because of associated anatomical variations of main bile duct and hepatic artery and increased risk of common bile duct injury.

## 1. Introduction

Gallbladder duplication is a rare congenital malformation, occurring in about one per 4000 births [1]. Congenital anomalies of the gallbladder and anatomical variations of their positions are associated with an increased risk of complications after laparoscopic cholecystectomy [2–5]. Preoperative imaging should be helpful for diagnosis. Laparoscopic removal of both gallbladders with intraoperative cholangiography seems to be the appropriate treatment.

## 2. Case Report

**Figure 1:**
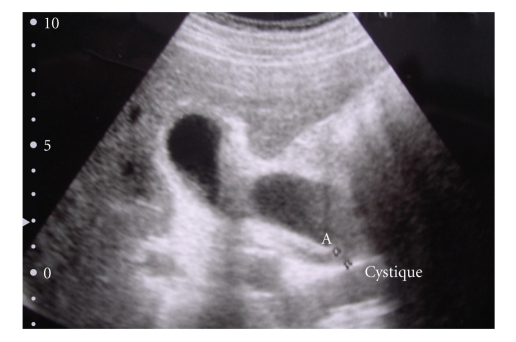
US findings showing a “bilobar” gallbladder.

**Figure 2:**
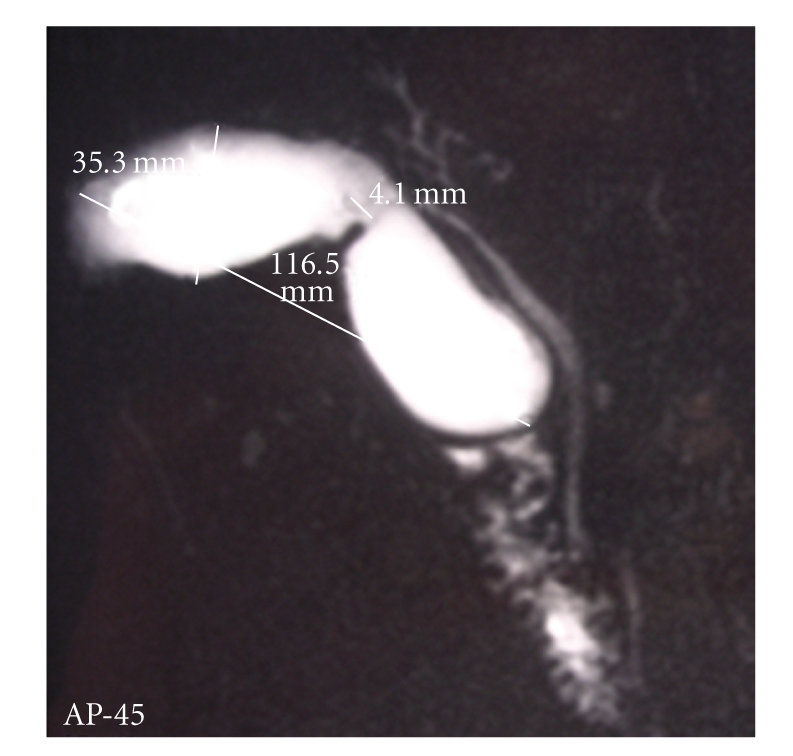
MRI findings. The cystic duct was not seen.

**Figure 3:**
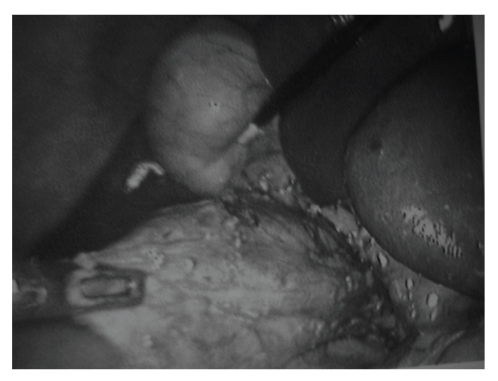
Perioperative view showing a true duplication of the gallbladder.

A 61-year-old man presented with epigastric and right upper quadrant pain associated with fever, nausea, and jaundice. Physical examination showed slight tenderness in the right upper quadrant. Laboratory values found elevated white blood cell count and C reactive protein associated with elevation of total bilirubin, transaminases and alkaline phosphatase. Angiocholitis was suspected. US examination of the right upper quadrant was performed (Figure 1). The gallbladder was tensely distended with wall thickening, presence of gallstone, and sonographic Murphy sign but no dilatation of bile duct. A MR cholangiography was also performed, showing a bilobar gallbladder with intravesicular stone and no stone in the common bile duct (Figure 2). The cystic duct was not described. No preoperative ERCP was performed. The patient received medical therapy with antibiotics and poor fat diet. Because of a mania attack the patient was hospitalized during 6 weeks in psychiatry unit and lost to follow-up. Five months later, the patient presented a new episode of acute cholecystis which was treated by intravenous antibiotics. The patient underwent laparoscopic cholecystectomy. At exploration, a duplication of the gallbladder was confirmed as a Y-shaped type (*vesica fellea duplex*). The both gallbladders were removed during the same procedure (Figure 3). Intraoperative cholangiography showed no common bile duct injury, but suspicion of bile-duct stone. Transcystic drainage tube was used and the patient underwent a postoperative ERCP to extract this bile-duct stone. The patient presented a postoperative abscess in the liver bed treated by CT-guided drainage. At the end of follow-up, (24 months later) the patient was alive and asymptomatic.

## 3. Discussion

**Figure 4:**
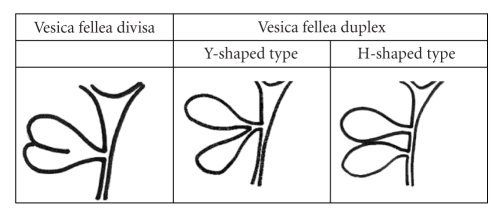
Boyden's classification of gallbladder duplication.

Duplication of the gallbladder is a rare congenital anomaly, occurring in about one per 4000 births [1]. It is thought to be due to exuberant budding of the developing biliary tree when the caudal bud of the hepatic diverticulum divides [6, 7]. The first reported human case was noted in a sacrificial victim of Emperor Augustus in 31 BC [5]. Because of associated anatomical variations of cystic duct and hepatic artery, this congenital anomaly is important to know for surgeons [5]. Senecail et al. found morphologic variations and abnormalities in more than 33% but only 3 cases of real duplication from ultrasonographic exploration of the gallbladder performed on 1823 patients [8]. Anatomic variants of gallbladder duplication are still differentiated according to Boyden's classification as follows (Figure 4) [1, 6]:

1. Vesica fellea divisa (bilobed or bifid gallbladder, double gallbladder with a common neck),
2. Vesica fellea duplex (double gallbladder with two cystic ducts),
  - Y-shaped type (the two cystic ducts uniting before entering the common bile duct),
  - H-shaped type (ductular type, the two cystic ducts entering separately into the biliary tree).

Differential diagnosis includes gallbladder diverticula, gallbladder fold, Phrygian cap, choledocal cyst, pericholecystic fluid, focal adenomyomatosis, and intraperitoneal fibrous bands [2]. The incidence and nature of clinical problems associated with duplicated gallbladder are similar to those encountered in the single viscus, including acute or chronic cholecystis, cholelithiasis, empyema, torsion, cholecystocolic fistula, lump in the abdomen, and carcinoma. There are no specific symptoms attributable to a double gallbladder. Simultaneaous removal of both gallbladders at surgery is recommended to avoid cholecystis and symptomatic gallstones in the remaining organ [2, 9]. Several publications reported successful laparoscopic cholecystectomy for a duplicate gallbladder [2, 4, 9–17].Schroeder and Draper reported a successful laparoscopic cholecystectomy for a tripe gallbladder [18]. Because there does not seem to be a significantly increased risk for subsequent disease, prophylactic cholecystectomy in an asymptomatic patient with gallbladder duplication is not recommended [2]. It could now be detected preoperatively by imaging studies. US is generally the first choice of imaging modality in patients with suspected biliary disease. US may diagnose gallbladder duplication if the viscera are located separately. Some criteria have been defined to diagnose gallbladder duplication on US examination in limited case reports [19–22]. Although US findings may suggest a double gallbladder, the cystic duct is usually not identified and it is often impossible, as in our case, to distinguish bilobed gallbladder from a true duplication by US. Duplication should be considered when two cystic ducts are present on preoperative imaging. MR Cholangiography proved to be a valid, noninvasive imaging technique for the evaluation of patients with suspected anomalies of the gallbladder after initial scanning with US [23]. Helical CT scan can also be helpful [22]. Duplication of the gallbladder has been detected by oral cholecystography, scintigraphy, and percutaneous transhepatic cholangiography but these examinations are not routinely used in patients with biliary disease [23]. Concomitance with other congenital anomalies, such as an anomalous right hepatic artery, has been described and may lead to intraoperative injury [5]. Attention is being focused on the need of complete evaluation during surgery by intraoperative cholangiography to prevent inadvertent injury to the biliary system [2].

Duplication of the gallbladder is a rare congenital abnormality, which requires special attention to the biliary ductal and arterial anatomy. Laparoscopic cholecystectomy with intraoperative cholangiography seems to be the appropriate treatment.
